# Complete mitochondrial genome sequence of giant tiger prawn, *Penaeus monodon*, of Bangladesh

**DOI:** 10.1128/mra.00118-25

**Published:** 2025-04-04

**Authors:** Dipta Chandra Pal, Shakila Nargis Khan, Muhammad Manjurul Karim

**Affiliations:** 1 Department of Microbiology, University of Dhaka 95324, Dhaka, Bangladesh; Rochester Institute of Technology, Rochester, New York, USA

**Keywords:** *Penaeus monodon*, giant tiger prawn, mitochondrial genome, D-loop

## Abstract

We report the complete mitochondrial genome sequence of the giant tiger prawn (*Penaeus monodon*) from Bangladesh. The circular genome spans 15,979 base pairs, with a GC content of 29.04%, and encodes 13 protein-coding genes, 22 tRNAs, 2 rRNAs, and 1 control region.

## ANNOUNCEMENT

The giant tiger prawn (*Penaeus monodon*), locally known as Bagda shrimp in Bangladesh, is a brackish water species of economic significance. Belonging to the family Penaeidae, it contributes approximately USD 407.25 million annually to Bangladesh’s revenue, accounting for 1.76% of global shrimp exports in 2021–2022 (1). However, disease outbreaks caused by viral and bacterial pathogens severely impact production, posing challenges to farmers and the economy (2, 3).

Understanding the genetic variation and population structure of *P. monodon* is crucial for biodiversity conservation and sustainable aquaculture (4). Molecular genetic studies in Bangladeshi populations remain limited, necessitating efforts to fill this gap. This study presents the complete mitochondrial genome of *P. monodon* from Bangladesh.

A specimen was collected from the Bhola River (22°40′44.66″N, 90°36′22.92″E) and immediately preserved at −26°C until DNA extraction. Mitochondria were isolated from telson tissue (0.1 g) following the Wieckowski protocol with modifications (5, 6). Telson tissue was homogenized using a precooled mortar and pestle. The homogenate was differentially centrifuged at 4°C to obtain the mitochondrial pellet. DNA was extracted using proteinase K digestion, phenol-chloroform purification, and ethanol precipitation (7). The quality of the extracted DNA was evaluated by gel electrophoresis on a 0.8% agarose followed by spectrophotometric analysis using a Colibri-Model LB 915 microvolume spectrophotometer (Berthold Technologies GmbH & Co. KG, Germany) to measure DNA purity and concentration. The presence of mt-DNA was confirmed via COI gene-specific PCR. NGS library preparation was performed using the Illumina DNA Prep kit (Illumina, San Diego, CA, USA). Sequencing was carried out on the Illumina NextSeq 550 platform, generating paired-end reads of 151 bp in length. A total of 107,508 raw reads were generated, corresponding to 23.9 Mbp of sequencing data with an average coverage depth of 45×. The raw data quality was checked using FastQC v0.12.1 (8). Quality filtering (Q <20) was performed with Trimmomatic v0.39 (9), and *de novo* assembly was conducted using SPAdes v3.15.5 (10), yielding a 15,979 bp contig mitochondrial genome.

Gene annotation was performed using MITOS v1.1.7 with the Invertebrate Mitochondrial Code (transl_table = 5) (11). Manual curation refined gene boundaries through NCBI BLAST comparisons with reference mitochondrial genomes of *P. monodon* (NC_002184.1, MN057663.1). The assembled genome showed 92.97% and 93.13% identity to these previously identified circular mitochondrial genomes, respectively.

**Fig 1:**
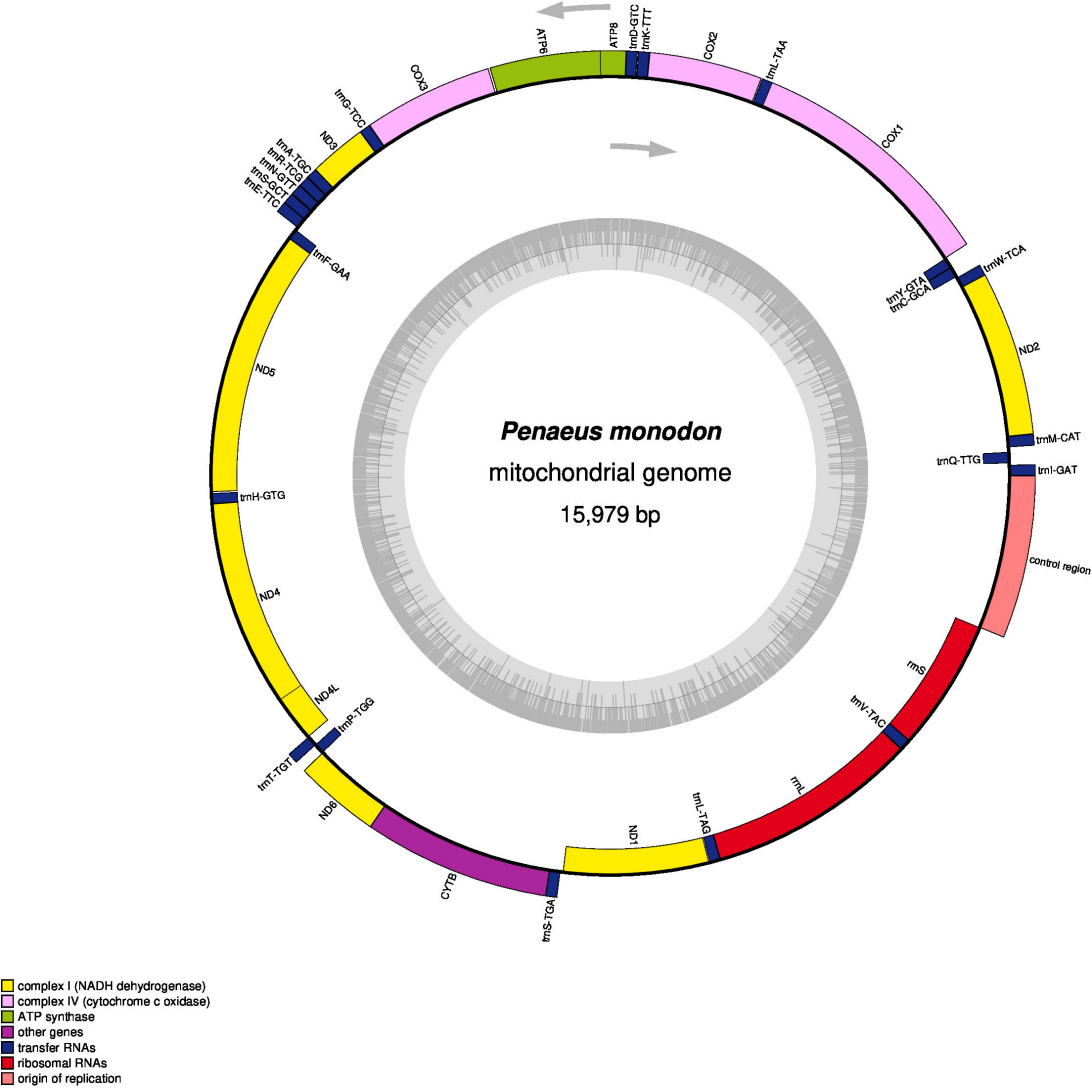
The mitochondrial genome map of *Penaeus monodon***.** It shows GC content in the innermost ring (ash grey), transcription directions as arrows, with clockwise-transcribed genes on the inner side and counterclockwise-transcribed genes on the outer side, color-coded gene groups (key in the bottom left corner) including 13 protein-coding genes (PCGs), 2 ribosomal RNA genes (*rrnS* for 12S and *rrnL* for 16S rRNA), 22 transfer RNA (tRNA) genes, and the putative control region.

The mitochondrial genome comprises a D-loop control region, 2 rRNA genes (12S and 16S), 22 tRNA genes, and 13 protein-coding genes. Of these, 23 genes are encoded on the heavy strand, and 14 on the light strand (Table 1). The nucleotide composition of the heavy strand includes 5,661 A (35.43%), 5,677 T (35.53%), 2,663 C (16.67%), and 1,978 G (12.38%). The ATP6, ATP8, COI-III, CYTB, ND2, ND3, and ND6 genes are encoded by the heavy strand, while the light strand encodes ND1, ND4, ND4L, and ND5. Visualization using OGDRAW generated a genome map (Fig. 1), showing transcription directions, gene locations, and GC content (12).

**TABLE 1:** Mitochondrial genome content, organization, and codon information of *P. monodon^a^*

Gene	Location	Gene length (bp)	Start codon	Stop codon	Anti-codon	H/L strand^*b*^	Intergenic region length (bp)
trnI	1–68	68			GAT	+	0
trnQ	81–151	71			TTG	−	12
trnM	182–250	69			CAT	+	30
ND2	251–1252	1,002	ATT	TAA		+	0
trnW	1251–1319	69			TCA	+	−2
trnC	1328–1395	68			GCA	−	8
trnY	1397–1462	66			GTA	−	1
COX1	1465–3003	1,539	ACG	TAA		+	2
trnL	2999–3065	67			TAA	+	−5
COX2	3071–3758	688	ATG	T^a^		+	5
trnK	3759–3827	69			TTT	+	0
trnD	3831–3898	68			GTC	+	3
ATP8	3899–4057	159	ATT	TAA		+	0
ATP6	4051–4725	675	ATG	TAA		+	−7
COX3	4736–5525	790	ATG	T^a^		+	10
trnG	5526–5592	67			TCC	+	0
ND3	5593–5944	352	ATG	T^a^		+	0
trnA	5945–6009	65			TGC	+	0
trnR	6012–6076	65			TCG	+	2
trnN	6078–6144	67			GTT	+	1
trnS	6148–6214	67			GCT	+	3
trnE	6215–6283	69			TTC	+	0
trnF	6303–6369	67			GAA	−	19
ND5	6370–8092	1,723	ATA	T^a^		−	0
trnH	8102–8168	67			GTG	−	9
ND4	8169–9509	1,341	ATG	TAA		−	0
ND4L	9503–9802	300	ATG	TAA		−	−7
trnT	9805–9871	67			TGT	+	2
trnP	9872–9937	66			TGG	−	0
ND6	9939–10460	522	ATC	TAA		+	1
COB/ CYTB	10460–11596	1,137	ATG	TAG		+	−1
trnS	11595–11664	70			TGA	+	−2
ND1	11682–12620	939	ATA	TAG		−	17
trnL	12626–12694	69			TAG	−	5
rrnL	12695–14059	1,365				−	0
trnV	14063–14134	72			TAC	−	3
rrnS	14135–14987	853				−	0
D-loop	14988–15979	992				+	0

^*a*^ Truncated termination codon.

^*b*^ Heavy (H) strand = “+”, Light (L) strand = “-”.

This study provides the first complete mitochondrial genome of *P. monodon* from Bangladesh, contributing valuable data for genetic and evolutionary studies of this economically important species.

## Data Availability

The complete mitochondrial genome sequence of *Penaeus monodon* has been deposited in the DDBJ/ENA/GenBank database under accession number PQ876261. The version described in this manuscript is the first version, PQ876261.1. The raw sequencing data from this study have been deposited in the NCBI Sequence Read Archive (SRA) under the accession number SRR31966867 (BioProject accession number PRJNA1209381 and BioSample accession number SAMN46219673).

## References

[1] Alam SMN. 2024. Portraying the Bangladesh shrimp industry: a SWOT analysis. Sustainability 16:1290. doi:10.3390/su16031290

[2] Hasan MM, Hoque MN, Ahmed F, Haque MIM, Sultana M, Hossain MA. 2022. Circulating phylotypes of white spot syndrome virus in Bangladesh and their virulence. Microorganisms 10:191. doi:10.3390/microorganisms10010191 35056639 PMC8780693

[3] Hossain MMM, Farjana N, Afroz R, Saha PK, Roy HS, Rahman MA, Farid MA. 2023. Genes expression in Penaeus monodon of Bangladesh; challenged with AHPND-causing Vibrio parahaemolyticus. Fish Shellfish Immunol Rep 4:100092. doi:10.1016/j.fsirep.2023.100092 37091065 PMC10114510

[4] Vu NTT, Zenger KR, Silva CNS, Guppy JL, Jerry DR. 2021. Population structure, genetic connectivity, and signatures of local adaptation of the giant black tiger shrimp (Penaeus monodon) throughout the Indo-Pacific Region. Genome Biol Evol 13:evab214. doi:10.1093/gbe/evab214 34529049 PMC8495139

[5] Wieckowski M, Giorgi C, Lebiedzinska M, Duszynski J, Pinton P. 2009. Isolation of mitochondria-associated membranes and mitochondria from animal tissues and cells. Nat Protoc 4:1582–1590. doi:10.1038/nprot.2009.151 19816421

[6] Pal DC, Hasan MM, Khan SN, Karim MM. 2025. Complete mitochondrial genome sequence of giant freshwater prawn, Macrobrachium rosenbergii of Bangladesh. Microbiol Resour Announc 14:e0092424. doi:10.1128/mra.00924-24 39601589 PMC11737144

[7] Green RM, Sambrook J. 2012. Molecular cloning: a laboratory manual. 4th edition. Vol. 33. Cold Spring Harbor Laboratory Press.

[8] Andrews S. 2020. Babraham bioinformatics - FastQC a quality control tool for high throughput sequence data. https://www.bioinformatics.babraham.ac.uk/projects/fastqc/.

[9] Bolger AM, Lohse M, Usadel B. 2014. Trimmomatic: a flexible trimmer for Illumina sequence data. Bioinformatics 30:2114–2120. doi:10.1093/bioinformatics/btu170 24695404 PMC4103590

[10] Bankevich A, Nurk S, Antipov D, Gurevich AA, Dvorkin M, Kulikov AS, Lesin VM, Nikolenko SI, Pham S, Prjibelski AD, Pyshkin AV, Sirotkin AV, Vyahhi N, Tesler G, Alekseyev MA, Pevzner PA. 2012. SPAdes: A new genome assembly algorithm and its applications to single-cell sequencing. J Comput Biol 19:455–477. doi:10.1089/cmb.2012.0021 22506599 PMC3342519

[11] Bernt M, Donath A, Jühling F, Externbrink F, Florentz C, Fritzsch G, Pütz J, Middendorf M, Stadler PF. 2013. MITOS: improved de novo metazoan mitochondrial genome annotation. Mol Phylogenet Evol 69:313–319. doi:10.1016/j.ympev.2012.08.023 22982435

[12] Greiner S, Lehwark P, Bock R. 2019. OrganellarGenomeDRAW (OGDRAW) version 1.3.1: expanded toolkit for the graphical visualization of organellar genomes. Nucleic Acids Res 47:W59–W64. doi:10.1093/nar/gkz238 30949694 PMC6602502

